# Preliminary network analysis of suicide risk and psychosocial factors in Chinese adults and older adults with depression: a cross-sectional study

**DOI:** 10.3389/fpsyt.2025.1702031

**Published:** 2025-12-17

**Authors:** Zhiyong Li, Yuhang Fan, Chenyu Li, Ting Men, Yang Shen

**Affiliations:** 1 Department of Psychiatry, Chuiyangliu Hospital affiliated with Tsinghua University, Beijing, China; 2 Lynch School of Education and Human Development, Boston College, Massachusetts, MA, United States; 3 Peking University HuiLongGuan Clinical Medical School, Beijing HuiLongGuan Hospital, Beijing, China

**Keywords:** older adults population, depressive disorders, suicide risk, psychosocial factors, network analysis

## Abstract

**Background:**

The study aims to elucidate the influence of multiple psychosocial determinants on suicidal ideation in late-life depression and to uncover their underlying network of interactions.

**Methods:**

This study analyzed patients with depressive disorders. A total of 2,052 patients were included, comprising 1,296 adult group and 756 older adults group. Clinical assessments were conducted using HAMD-17, HAMA, SCL-90, PSQI and NGASR. Data analysis and visualization were performed using R software. The network structure among psychological variables was assessed, and the variable nodes that play a key role in the network were identified. Independent network modeling was conducted separately for the adult group and older adults group, so as to further examine the properties and differences of the network structure between two groups.

**Results:**

Depression emerged as the strongest predictor of suicidal ideation in adults, showing the highest degree of association. Anxiety demonstrated a dual role. Among older adults, the coupling between variables was stronger, particularly the combined effects of depression and sleep disturbances. The PSQI node exhibited more pronounced bridging properties in the older adults group, serving as the most significant mediator across the two network structures. The anxiety–depression pathway displayed greater connectivity in the older adults group, with anxiety exerting a stronger indirect effect on suicidal ideation.

**Conclusions:**

Depression is a key predictor of suicidal ideation, while anxiety exerts a dual influence. Suicidal ideation among older adults appears to be more strongly shaped by multiple factors, with particular emphasis on the combined effects of depression and sleep disturbances.

## Introduction

As the global population ages, mental health issues among the older adults have become more prevalent. Geriatric depression, a common mental disorder, severely threatens the physical and mental health, as well as the quality of life, of older adults (1). According to the statistics from World Health Organization (WHO), the suicide mortality rate among Chinese people aged 60 and above accounts for 7.2% of the total population, significantly higher than that of younger groups. There are differences in suicide rates among patients with depression from different ethnic groups. Patients in China may experience higher suicide rates due to delayed intervention caused by poorer access to medical resources and stronger stigma against mental illness within their culture, or due to facing greater cultural adaptation pressures (2). Among older adults individuals who have suicidal intent or have attempted suicide, 50-70% were diagnosed with major depressive disorder. In 2019, the prevalence of depression among the older adults population in China was approximately 3.8%. Individuals entering the older adulthood at the age of 65–69 significantly increased the risk of MDD by 64.9% (3). In this population, approximately 38.2% to 60% of individuals are at risk of suicide (4, 5). The suicide rate among older adults patients with depression was found to be at least twice as high as that of the general population, making suicidal behavior in this group a pressing public health concern that cannot be overlooked. However, in the older adults population of China, the recognition rate of depression is low, and the assessment of suicide risk is seriously inadequate (6).

Suicidal ideation, as a precursor to suicidal behavior, is an important indicator for predicting suicide risk. Among older adults patients with depression, the emergence of suicidal ideation is often the result of multiple interacting factors, with psychosocial elements playing a critical role in this process (7). There is a close connection between geriatric depression and suicide risk (8). Older adults patients with depression are more likely to have negative experiences due to declines in physiological functions, changes in social roles, and decreased psychological adaptability, which might increase their suicide risk. Research indicates that their suicide risk is 5 to 10 times higher than that of the general population. (9). Suicide risk serves as a crucial indicator for predicting suicidal behavior. In older adults patients with depression, this risk typically arises from the interplay of various factors, with psychosocial elements playing a significant role (10).Older adults patients with depressive disorders frequently experience negative emotions such as helplessness, hopelessness, and loneliness (11). In addition, they often experience significant role transitions and adverse life events, including widowhood, retirement, children leaving home, and the loss of close relatives (12), all of which can readily lead to mental health challenges. Moreover, with advancing age, physical functions gradually decline and chronic illnesses, such as hypertension, diabetes, and heart disease become more prevalent. The physical symptoms associated with these conditions, including pain, insomnia, and loss of appetite, further weaken patients’ psychological resilience and elevate their risk of suicide (13, 14).

At present, research on the relationship between suicidal ideation and psychosocial factors in older adults patients with depression has primarily focused on the analyses of single-factors or a limited set of variables, without addressing the potential complex interactions among them. According to the network theory in psychopathology, symptoms of mental disorders interact causally with one another. When these interactions are sufficiently strong, they form feedback loops that perpetuate the disorder (15). Network analysis, as a graph theory-based statistical modeling method, describes the relationships within a system by using nodes and edges (16, 17). In the field of psychiatric research, network analysis has been extensively applied to explore symptom interactions, comorbidity mechanisms, and the dynamic evolution of overall psychological states (18). However, studies using network analysis to investigate the relationship between suicidal ideation and psychosocial factors in Chinese older adults patients with depression remain limited.

The present study aims to employ network analysis to examine in depth the complex relationships between suicidal risk and psychosocial factors in Chinese older adults patients with depression. By identifying key factors and core nodes, the study seeks to uncover the interaction patterns among these elements, the study seeks to offer theoretical foundations and practical guidance for the precise assessment and effective intervention of suicide risk in this population. The ultimate goal is to reduce the suicide rates among older adults patients with depression, while enhancing their quality of life and mental well-being.

## 2 Materials and methods

### 2.1 Participants

This study retrospectively analyzed patients diagnosed with depressive disorders who were treated in the Department of Psychiatry at Chuiyangliu Hospital affiliated with Tsinghua University between August 2023 and August 2024. A total of 2052 cases were screened. The inclusion criteria were as follows: (1) age ≥ 18 years; (2) diagnosis of moderate/severe depressive episode or recurrent depressive disorder according to the diagnostic criteria of the *International Classification of Diseases, 10th Revision* (ICD-10); (3) score of 17-item Hamilton Depression Rating Scale (HAMD-17) > 7; (4) ④ score of Nurses’ Global Assessment of Suicide Risk (NGASR) ≥ 6, with the presence of suicidal ideation. Exclusion criteria included any of the following: (1) history of manic or hypomanic episodes; (2) history of dysthymia; (3) intellectual disability; (4) severe physical illness in the acute phase at the time of assessment; (5) exposure to major life events such as divorce, bereavement, etc.; ⑥ presence of physical disability or marked mobility limitation. Finally, a total of 2052 patients were included in this study, including 1,296 patients aged 18–60 years (adult group) and 756 patients aged ≥60 years (older adults group).

The study protocol and informed consent form have been approved by the Ethics Committee of Chuiyangliu Hospital Affiliated to Tsinghua University. Written informed consent was obtained from all participants or their legal guardians prior to study enrollment.

### 2.2 Clinical assessment

#### 2.2.1 Demographic data

This study examined sociodemographic variables, including gender, age, marital status, location of residence, and household income (19). Marital status was coded dichotomously as “with spouse” or “without spouse.” Residence was classified as urban or rural according to registered addresses. Family income was categorized relative to the local mean annual household income, with values above the mean defined as high income and those below defined as low income.

All eligible participants were assessed by two physicians supported by attending physicians. All evaluating physicians have undergone consistency evaluation training. This study selected tools such as HAMD-17, HAMA, SCL-90, PSQI, and NGASR to evaluate suicide risk and symptoms in patients with depression. The core reason for this selection is that these tools possess complementary multi-dimensional assessment capabilities. The results of the above scales can help comprehensively understand the psychological status, symptom characteristics, and suicide risk level of patients with depression.

#### 2.2.2 17-item Hamilton depression rating scale

The HAMD-17 (20), developed by Hamilton, is one of the most widely used instruments in clinical practice for evaluating depressive states. The HAMD-17 assesses depressive symptoms experienced during the preceding week and consists of 17 items. Each item is rated on a 5-point scale (0–4), where 0 indicates no symptoms, 1 mild, 2 moderate, 3 severe, and 4 extremely severe. Higher total scores reflect greater severity of depressive symptoms. A total score above 24 suggests severe depression, scores above 17 indicate mild to moderate depression, and scores below 7 suggest the absence of clinically significant depressive symptoms.

#### 2.2.3 Hamilton anxiety rating scale

The HAMA (21), developed by Hamilton in 1959, is one of the commonly used instruments in psychiatric clinical practice for assessing anxiety severity. It is primarily applied to patients with neuroses and related conditions but is not recommended for evaluating anxiety severity in individuals with psychotic disorders. The HAMA consists of 14 items, each rated on a 5-point scale (0-4), where 0 indicates no symptoms, 1 mild, 2 moderate, 3 severe, and 4 extremely severe. The total score of HAMA can effectively reflect the severity of anxiety symptoms and is used to evaluate the severity of anxiety in patients with anxiety and depressive disorders, as well as the effectiveness of various pharmacological and psychological interventions. According to data provided by the Chinese Scale Cooperative Group, a total score ≥29 indicates severe anxiety; ≥21 indicates marked anxiety; ≥14 indicates moderate anxiety; >7 suggests mild anxiety; and <7 indicates no clinically significant anxiety symptoms.

#### 2.2.4 Symptom checklist-90

The SCL-90 is a widely used mental health assessment instrument and is commonly employed as an outpatient screening tool for mental disorders (22). Developed by L.R. Derogatis in 1975, it is suitable for individuals aged 16 and above. The scale was introduced to China in the 1980s and translated into Chinese by Wang Zhengyu in 1984. The scale consists of 90 items, assessing a wide range of psychiatric symptoms, including sensations, emotions, thoughts, consciousness, behaviors, living habits, interpersonal relationships, diet, and sleep etc.

#### 2.2.5 Pittsburgh sleep quality index

The PSQI was developed by Buysse DJ and colleagues at the University of Pittsburgh Department of Psychiatry in 1989 (23). This scale is suitable for assessing sleep quality in individuals with sleep disorders, other psychiatric conditions, and in the general population. The PSQI consists of nine questions: the first four are fill-in-the-blank questions, and the last five are multiple-choice questions, with Question 5 containing 10 sub-items. The instrument is used to assess the subject’s sleep quality over the past month, comprising 19 self-rated items and five observer-rated items. Among these, the 19th self-rated item and all five observer-rated items are excluded from scoring; only the rest 18 self-rated items that contribute to the score are described here. These 18 items form 7 components, each scored on a 0–3 scale. The global PSQI score is the sum of the component scores, ranging from 0 to 21, with higher scores indicating poorer sleep quality.

#### 2.2.6 Nurses’ global assessment of suicide risk

The NGASR evaluates multiple factors that may contribute to suicide risk (24). Common assessment dimensions include: suicide risk, past suicidal behaviors, mental illness status, social support, life event stress, and substance abuse. In terms of scoring, the scale typically assigns scores based on responses to different questions, and the total score is used to determine the level of suicide risk. Generally, a higher score indicates a higher suicide risk. Responses indicating a clear suicide plan and intent to implement it are assigned higher scores, while responses indicating good social support and no suicide risk receive lower scores.

The NGASR exists in multiple versions, which may differ in content and scoring methods. In general, the scale is designed to evaluate a range of risk factors for suicide. Common assessment dimensions include suicidal ideation, history of suicidal behavior, psychiatric status, level of social support, stress from life events, and substance abuse. Scoring is typically based on assigning values to responses for each item, with the total score used to determine the overall level of suicide risk. Higher scores correspond to greater suicide risk. Responses indicating the presence of a clear suicide plan and intent to act are assigned higher scores, whereas those reflecting strong social support and the absence of suicidal ideation receive lower scores. Ultimately, professionals interpret NGASR scores in conjunction with clinical judgment and the individual’s circumstances to determine the degree of suicide risk and to formulate appropriate interventions.

### 2.3 Statistical analysis

Demographic data were analyzed using SPSS version 30.0. The chi-square (χ²) test was employed to compare differences between the non-suicidal ideation group and the suicidal ideation group with respect to gender, age, marital status, years of education, location of residence (rural vs. urban), and annual household income. The sample size is determined through a validated formula, referencing the expected prevalence of suicide risk, with a 5% margin of error and 95% confidence level set. A two-tailed significance level of 0.05 was applied.2.3.2 Network Analysis.

Psychological network analysis, through the ‘symptom connection perspective’, can accurately capture the differences in risk pathways and core nodes between the two groups, providing a more scientific basis for stratified assessment and precise intervention of suicide risk in adult and older adults patients. R software (V.4.4.3) was used for data analysis and graph visualization (25). The network structure among psychological variables was estimated using the EBICglasso algorithm (a combination of graphical LASSO and Extended Bayesian Information Criterion) to capture sparse partial correlations among variables.

After network modeling, three centrality indices for each node were further calculated: node strength, expected influence, and closeness centrality. These indices were visualized and ranked using the centralityPlot() function to identify variable nodes that play key roles in the network.

To evaluate the confidence of the network structure and the stability of edge weight estimates, bootstrapping was performed with 1,000 repeated samplings to analyze the confidence intervals and significance of differences for each edge weight and centrality index. Meanwhile, the “case-dropping bootstrap” method was used to test the stability of centrality indices, and the CS-Correlation Stability coefficient was calculated using the corStability() function. According to recommended standards, a CS coefficient greater than 0.25 is acceptable, and a value exceeding 0.50 indicates high stability (26).

For inter-group comparisons, samples were divided into two groups based on age: the adult group (18–59 years) and the older adults group (≥60 years). The above network modeling and index calculation procedures were performed independently for each group.

To further test whether there were significant differences in network structure between the different age groups, the Network Comparison Test (NCT) method in the NetworkComparisonTest package was used, implementing the following three tests: (1) Network Structure Invariance test (for overall differences in network structure); (2) Global Strength Invariance test (for differences in overall network strength); (3) Edge Invariance test (for item-by-item differences in edge weights).

The permutation test was set to 1,000 iterations, and the Benjamini-Hochberg method was used to correct p-values for multiple comparisons of edge weight differences. The analysis results showed that there were statistically significant differences in the weights of several edges between the adult group and the older adults group (see Results section for details), suggesting potential heterogeneity in the association structure between psychological variables across different age groups (27).

## 3 Results

### 3.1 Demographic characteristics and clinical assessment results of the two groups

A total of 1277 participants were included in the adult group (423 men and 854 women), and 746 participants were included in the older adults group (239 men and 507 women). In terms of suicide risk scores, the suicide risk score of the older adults group was significantly higher than that of the adult group (p < 0.01). Regarding psychiatric symptoms, the scores of anxiety, depression, and other psychological symptoms in the older adults group were significantly lower than those in the adult group, while the sleep quality score was significantly higher in the older adults group (p < 0.01). The proportion of participants without spouses in the older adults group was significantly higher than that in the adult group (p < 0.01). There were no statistically significant differences between the two groups in terms of gender, place of residence, or family income. See **Table 1** for details.

**Table 1:** Demographic characteristics and scale assessment results of the two groups.

Variables	Older adults group (n=746)	Adult group (n=1277)	*t/χ² value*	*p-value*
Gender (M/F)	239/507	423/854	0.253	0.615
Marital status (with spouse/without spouse)	393/353	804/437	28.507	<0.01*
Residence (urban/rural)	498/248	877/400	0.798	0.372
Family income (high/low)	279/467	451/826	0.885	0.347
HAMD	13.71 ± 6.55	17.01 ± 8.68	-10.990	<0.01*
HAMA	16.86 ± 6.19	18.42 ± 7.86	-5.358	<0.01*
SCL90	1.65 ± 0.45	2.35 ± 0.93	-25.442	<0.01*
PSQI	12.76 ± 3.96	11.45 ± 4.55	7.372	<0.01*
NGASR	11.08 ± 6.25	9.55 ± 4.93	8.001	<0.01*

M: Male; F: Female; HAMD: Hamilton Depression Rating Scale; HAMA: Hamilton Anxiety Rating Scale; SCL-90: Symptom Checklist-90; NGASR: Nurses’ Global Assessment of Suicide Risk; PSQI: Pittsburgh Sleep Quality Index. *p < 0.05 indicates statistical significance.

### 3.2 Network analysis of suicide risk and psychiatric symptoms in the adult group

**Figure 1:**
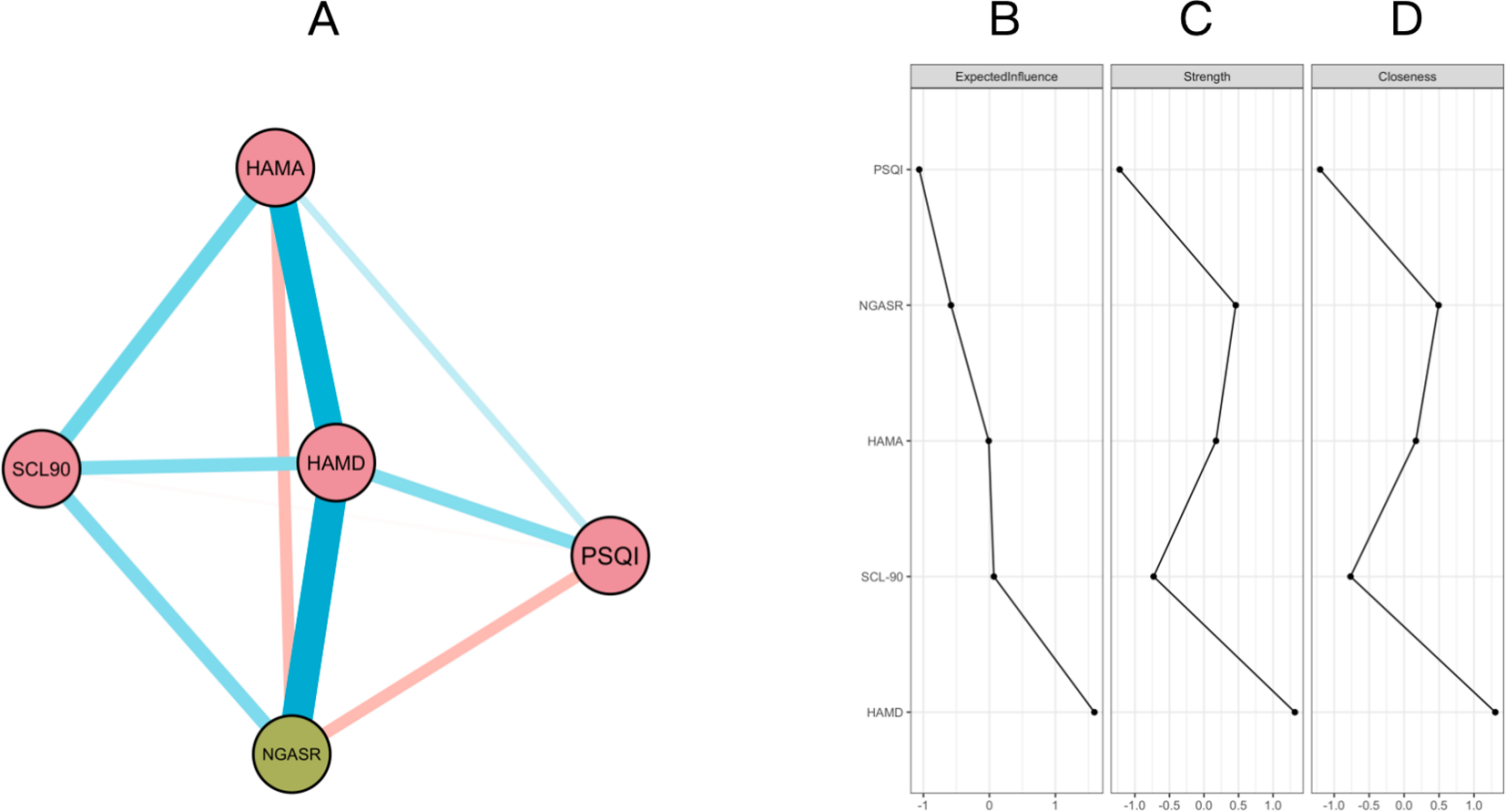
**(A)** Network structure diagram of psychological variables related to depression, anxiety, sleep, and suicide risk in the adult group. Nodes represent the total scores of scales, including SCL-90 (Symptom Checklist-90), HAMA (Hamilton Anxiety Scale), HAMD (Hamilton Depression Rating Scale), PSQI (Pittsburgh Sleep Quality Index), and NGASR (Nurses’ Global Assessment of Suicide Risk). The color of nodes indicates the type of variables, among which green nodes represent suicide risk and red nodes represent other psychological symptoms. The color of edges indicates the direction of correlation between variables: blue edges represent positive correlations, and red edges represent negative correlations. The thickness of edges reflects the strength of correlation; the thicker the edge, the stronger the connection between two variables. **(B)** Expected Influence of each node, reflecting the overall impact degree of the node in the network; **(C)** Strength of the node, that is, the sum of the connection strengths between a node and other nodes; **(D)** Closeness centrality of the node, representing the reciprocal of the average path length between the node and other nodes in the network.

In the network graph, nodes represent the total scores of each scale, and edges reflect conditional correlations between nodes; the thickness of edges indicates the strength of association, and colors distinguish positive (blue) and negative (red) correlations. As shown in the network structure **Figure 1**, the network illustrating the relationships between the scores of various scales contains 10 regularized partial correlations, among which 7 edges are positive correlations and 2 edges are negative correlations. Suicide risk has the strongest association with HAMD scores. The depression node has the highest closeness centrality, expected influence, and strength centrality, suggesting that depression scores are a direct predictor of suicide risk. Secondly, there is a correlation between PSQI and suicide risk, and the PSQI node has a moderate-weight impact on suicide risk, indicating that sleep problems can affect the formation and disappearance of suicide risk. The SCL-90 node has the weakest impact on suicide risk, suggesting that its predictive effect on suicide risk is poor. In addition, anxiety has a dual impact on suicide risk. There is a direct and strong negative correlation between anxiety and suicide risk, indicating that increased anxiety can inhibit the occurrence of suicide risk. However, the anxiety node has a higher positive correlation with the depression node, which can indirectly strengthen the occurrence of suicide risk through the depression node. Therefore, it is suggested that anxiety may play a certain regulatory role between depression and suicide risk.

The above results indicate that depression is an important predictor of suicide risk in adults, while anxiety has a dual impact on suicide risk.

### 3.3 Network analysis of suicide risk and psychiatric symptoms in the older adults group

**Figure 2:**
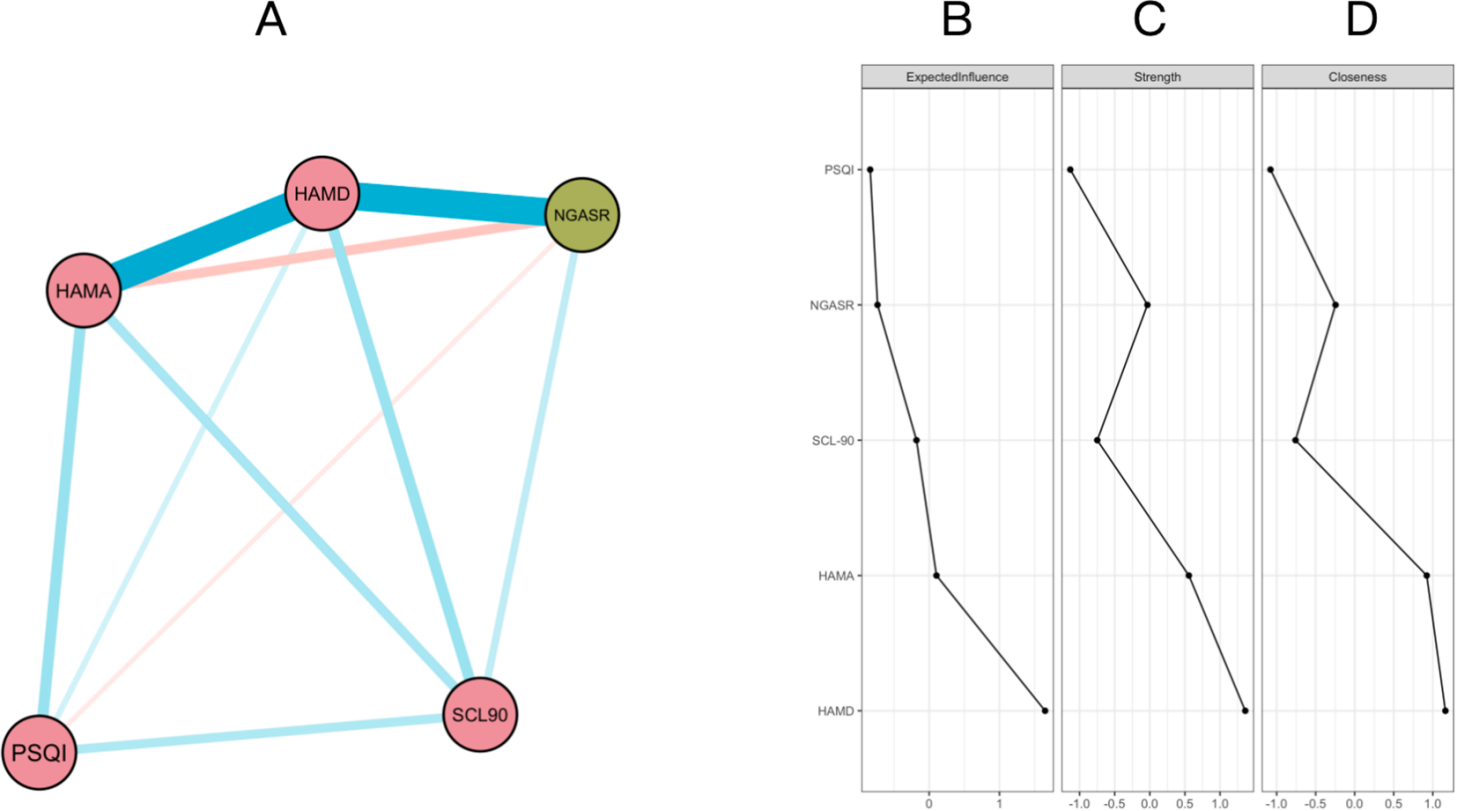
**(A)** Network structure diagram of psychological variables related to depression, anxiety, sleep, and suicide risk in the older adults group. Nodes represent the total scores of scales, including SCL-90 (Symptom Checklist-90), HAMA (Hamilton Anxiety Scale), HAMD (Hamilton Depression Rating Scale), PSQI (Pittsburgh Sleep Quality Index), and NGASR (Nurses’ Global Assessment of Suicide Risk). The color of nodes indicates the type of variables, among which green nodes represent suicide risk and red nodes represent other psychological symptoms. The color of edges indicates the direction of correlation between variables: blue edges represent positive correlations, and red edges represent negative correlations. The thickness of edges reflects the strength of correlation; the thicker the edge, the stronger the connection between two variables. **(B)** Expected Influence of each node, reflecting the overall impact degree of the node in the network; **(C)** Strength of the node, i.e., the sum of the connection strengths between a node and other nodes; **(D)** Closeness centrality of the node, representing the reciprocal of the average path length between the node and other nodes in the network.

As shown in the network structure **Figure 2**, the network illustrating the relationships between the scores of various scales contains 9 regularized partial correlations, among which 7 edges are positive correlations and 2 edges are negative correlations. The depression node still has the highest correlation with suicide risk, which is consistent with the situation in the adult group. However, in the older adults group, the correlation between PSQI and suicide risk is enhanced, showing an obvious negative correlation. This suggests that the combined assessment of depression and sleep in the older adults group plays a stronger role in predicting suicide risk, indicating that therapists need to pay more active attention to sleep problems in the older adults population. The SCL-90 node in the older adults group has an obvious connection with suicide risk, indicating that the overall level of psychological distress in the older adults has a more direct impact on suicide risk.

The above results suggest that the coupling between variables is stronger in the older adults group, and suicide risk may be affected by multiple factors simultaneously, especially the superimposed effect of depression and sleep problems.

### 3.4 Differences in the correlation between suicide risk and psychological symptoms between the older adults group and the adult group

In the older adults group, the correlation of PSQI with more factors increased, including SCL-90 (p=0.001), suicide risk (p=0.002), HAMA (p=0.040), and HAMD (p=0.002), with significant differences between the two age groups. This suggests that the PSQI node in the older adults group has more prominent characteristics as a bridge variable and is the mediating node with the most significant difference in the inter-group network structure.

In the older adults group, the correlations of SCL-90 (p=0.021) and PSQI (p=0.002) with suicide risk were higher than those in the adult group, while HAMD (p=0.842) and HAMA (p=0.592) showed no statistical differences between the two groups. In the older adults population, suicide risk is more susceptible to the impact of sleep disorders and overall psychological distress.

The connection strength of the anxiety-depression pathway in the older adults group was significantly higher than that in the adult group (p=0.001). This indicates that the anxiety-depression pathway has stronger linkage in the older adults, and anxiety has a higher intensity of indirect impact on suicide risk.

To explore the stability of the network structure among psychological variables across different groups (or conditions), this study conducted edge weight difference tests on the network structure between SCL-90, HAMA, HAMD, PSQI, and NGASR. See **Table 2**.

**Table 2:** Analysis of differences in network structure edge weights between psychological variables in the two groups.

Variable pairing	Raw *p-value*	Test statistic E	Adjusted *p-value*
HAMA-HAMD	0.001*	0.098	0.02*
SCL90-PSQI	0.001*	0.188	0.02*
HAMD-PSQI	0.002*	0.141	0.02*
PSQI-NGASR	0.002*	0.142	0.02*

*p < 0.05 indicates statistical significance.

The results showed that there were significant differences in a total of four edges between the networks of the two groups (all adjusted p-values = 0.02), as follows: The edge weight difference between HAMA and HAMD was significant (E = 0.0980, p= 0.001), indicating that there was a statistical difference in the association degree between anxiety and depression among different groups.

The edge difference between SCL-90 and PSQI was significant (E = 0.1883, p= 0.001), suggesting that the connection between the overall psychological symptom burden and sleep quality differed between the two groups.

The edge difference between HAMD and PSQI was significant (E = 0.1406, p= 0.002), indicating that the connection strength between depressive symptoms and sleep problems varied under different conditions.

There was also a significant difference in the edge weight between PSQI and NGASR (E = 0.1416, p=0.002), suggesting that the relationship between sleep disorders and suicide risk had different structural characteristics across groups.

In summary, these results indicate that the connections between the HAMA node, HAMD node, PSQI node, and NGASR node are variable across different groups or conditions. In particular, sleep, as a key variable, shows structural differences in its relationship with multiple psychological symptoms across different networks. The above findings emphasize that in psychological intervention practices, it is necessary to combine specific population characteristics to accurately identify key connection paths.

## 4 Discussion

This study reveals that depression is the most significant predictor of suicide risk among older adults patients with depression, aligning closely with global research findings. Numerous domestic and international epidemiological surveys indicate a strong positive correlation between the severity of depressive symptoms and the level of suicide risk (28). Especially in the older adults population, depression has been widely verified as the “core driving factor” of suicidal behavior (29). This finding further supports the stability of the ‘depression-suicide risk’ pathway among the older adults, indicating that in clinical settings, monitoring depressive symptoms should be prioritized when evaluating suicide risk in older adults patients with depression.

Anxiety has a dual impact on suicide risk. It can both directly decrease suicide risk and indirectly increase it by mediating depression. This offers a new perspective on the intricate relationship between anxiety and suicide. Traditionally, anxiety has been viewed as a standalone risk factor for suicide, but this research reveals its contradictory nature. This duality likely arises from anxiety’s dual characteristics: “adaptive” and “pathological.” On one hand, moderate anxiety may encourage individuals to seek help, thus directly lowering immediate suicide risk (30). On the other hand, long-term anxiety exacerbates depressive symptoms by overactivating the HPA axis, creating a vicious ‘anxiety-depression’ cycle that indirectly raises the risk of suicide (31). This finding aligns with the ‘double-edged sword effect of anxiety’ theory, which posits that in clinical interventions, it is crucial to differentiate between various pathways of anxiety and avoid one-sided approaches (32).

This study also found that the coupling intensity of various psychosocial variables among the older adults is significantly higher than in adults. The combined effect of depression and sleep problems has the most pronounced impact on suicide risk. This finding supports the ‘multi-factor interaction model’ of older adults mental health. As individuals age, they encounter multiple challenges, including declines in physical function, loss of social roles, and the accumulation of chronic illnesses. Social isolation is an important factor that exacerbates the risk of suicide among the older adults, with its effects primarily transmitted through three dimensions: lack of emotional support, negation of self-worth, and obstacles to accessing survival resources. If the older adults remain in a state of social isolation for a long time, they lose emotional connections provided by family, friends, or the community. When facing life stressors such as illness, bereavement, or retirement, they lack channels for venting and emotional buffering, leading to the accumulation of persistent feelings of loneliness and helplessness. Meanwhile, prolonged disconnection from social interaction weakens their perception of self-worth. Some older adults may develop a negative judgment that ‘living is meaningless’ due to the cognition of ‘being unneeded’, gradually progressing to a sense of hopelessness. This constitutes the core psychological basis for the formation of suicidal ideation. The influence of religious belief on the risk of suicide among the older adults is also dual in nature. On one hand, religious communities can provide mutual support to alleviate feelings of loneliness, thereby reducing the risk of suicide. On the other hand, some elderly people, due to their one-sided interpretation of religious doctrines, may overly rely on religious groups when facing difficulties, while neglecting effective intervention methods. As a result, they indirectly increase the risk of suicide (33, 34). These factors make psychosocial influences more likely to create a ‘linkage effect’ (35).

The incidence of sleep disorders among older adults patients with depression is over 70%. Sleep deprivation can further impair emotional regulation and exacerbate depressive symptoms (36). The two reinforce each other to form a closed loop, eventually sharply increasing the suicide risk. This study is the first to elucidate the characteristics of the PSQI node as a bridge variable in the older adults population. As the mediating node with the most significant intergroup differences, it highlights the ‘hub’ status of sleep disorders within the older adults suicide risk network (37). Sleep disorders are not only common accompanying symptoms of depression and anxiety, but also independent factors that exacerbate psychological distress. In the older adults population, sleep problems, by affecting cognitive functions and emotional regulation, become the key intermediary connecting various psychosocial problems and suicide risk (38, 39). This aligns with the discussion of the ‘sleep-psychology-behavior’ triad in geriatric research, indicating that enhancing sleep quality could be a breakthrough intervention target for reducing suicide risk among the older adults.

Based on the findings of this study, we offer the following recommendations for treating older adults patients with depression. First, depression symptoms should be considered the primary indicator for assessing suicide risk, with their severity regularly evaluated. Second, anxiety symptoms should be managed individually, with timely and appropriate interventions to prevent exacerbation of depression. Third, prioritize sleep management and use the PSQI score as a key supplementary indicator for risk assessment. Finally, for older adults patients, implement ‘multi-target combined intervention’ to address depression, anxiety, and sleep issues simultaneously.

This study has several limitations. First, its cross-sectional design prevents the clarification of causal relationships between variables. Although this study did not determine whether there is a causal relationship between each research factor and suicide risk, it has laid a foundation for further research on suicide issues in older adults patients with depression. Future research should employ longitudinal tracking to examine the dynamic evolution of the ‘anxiety-depression-sleep’ pathway and observe symptom changes. Second, the sample is limited to a specific region, potentially introducing regional bias. In future research, we can further expand the sample size, conduct multi-center and multi-regional collaborative projects, increase longitudinal follow-up, to clarify the relationship between various factors and suicide risk in older adults patients with depression. Additionally, future research could focus on longitudinal data to develop a dynamic network model that captures the evolving patterns of suicide risk over time. Intervention studies could then be conducted to assess the effectiveness of targeted interventions, particularly those addressing key factors like depression and sleep, in mitigating suicide risk.

## 5 Conclusions

The HAMD score is a crucial predictor of suicide risk in adults, showing a strong correlation with this risk. Similarly, the HAMA score influences suicide risk both directly and indirectly. In the older adults, suicide risk is influenced by various factors, with the HAMD and PSQI scores having a combined effect. Therefore, it is essential to monitor emotional and sleep issues in older adults closely.

## Data Availability

The raw data supporting the conclusions of this article will be made available by the authors, without undue reservation.
